# Increased Outbreaks Associated with Nonpasteurized Milk, United States, 2007–2012

**DOI:** 10.3201/eid2101.140447

**Published:** 2015-01

**Authors:** Elisabeth A. Mungai, Casey Barton Behravesh, L. Hannah Gould

**Affiliations:** Atlanta Research and Education Foundation, Atlanta, Georgia, USA (E.A. Mungai);; Centers for Disease Control and Prevention, Atlanta (E.A. Mungai, C.B. Behravesh, L.H. Gould)

**Keywords:** Unpasteurized milk, nonpasteurized, dairy products, *E. coli*, *Campylobacter*, bacteria, United States

## Abstract

The number of US outbreaks caused by nonpasteurized milk increased from 30 during 2007–2009 to 51 during 2010–2012. Most outbreaks were caused by *Campylobacter* spp. (77%) and by nonpasteurized milk purchased from states in which nonpasteurized milk sale was legal (81%). Regulations to prevent distribution of nonpasteurized milk should be enforced.

Pasteurization is an effective way to improve milk safety; however, in the United States, illness related to consumption of nonpasteurized milk continues to be a public health problem. The first statewide requirements that dairy products be pasteurized were enacted in Michigan in 1948 (*1*). In 1987, the US Food and Drug Administration banned the interstate sale or distribution of nonpasteurized milk. However, the laws regulating intrastate sales are set by each state (*2*). Regulations for intrastate sales of nonpasteurized milk vary from complete bans to permitting sales from farms or retail outlets (*2*). Even in states in which sale of nonpasteurized milk is illegal, milk can often be obtained through other means. For example, some states allow cow-share or herd-share agreements, in which buyers pay farmers a fee for the care of a cow in exchange for a percentage of the milk produced (*3*,*4*).

Consumption of nonpasteurized milk has been associated with serious illnesses caused by several pathogens, including *Campylobacter* spp.*,* Shiga toxin–producing *Escherichia coli,* and *Salmonella enterica* serotype Typhimurium (*3*,*4*). Despite the health risks associated with consuming nonpasteurized milk, the demand for nonpasteurized milk has increased (*3*,*5*,*6*). Recently, many state legislatures have considered relaxing restrictions on the sale of nonpasteurized milk (*2*,*6*). We report that the number of outbreaks associated with nonpasteurized milk increased from 2007 through 2012.

## The Study

A foodborne disease outbreak is defined as the occurrence of >2 cases of a similar illness resulting from ingestion of a common food. State and local health departments voluntarily report outbreaks to the Foodborne Disease Outbreak Surveillance System of the Centers for Disease Control and Prevention through a standard web-based form (www.cdc.gov/nors). We reviewed outbreaks reported during 2007–2012 in which the food vehicle was nonpasteurized milk. Outbreaks attributed to consumption of other dairy products made with nonpasteurized milk, such as cheese, were excluded. We analyzed outbreak frequency, number of illnesses, outcomes (hospitalization, death), pathogens, and age groups of patients. Data on the legal status of nonpasteurized milk sales in each state were obtained from the National Association of State Departments of Agriculture (*7*–*9*) and an online search of state regulations. The sources from which nonpasteurized milk was obtained or purchased were categorized according to the description from the state outbreak reports, when available.

**Figure 1:**
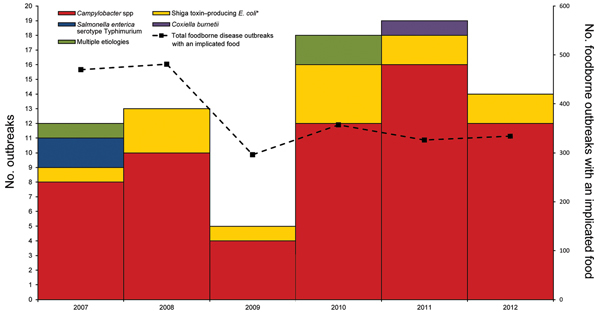
Outbreaks associated with nonpasteurized milk, by etiologic agent and year, United States, 2007–2012. Three outbreaks involved multiple pathogens: *Campylobacter* spp. and *Salmonella* *enterica* serotype Typhimurium; Shiga toxin–producing *Escherichia coli* O157:H7 and *Campylobacter;* *Campylobacter* and *Cryptosporidium*. *E. coli* serogroups: O157 (10 outbreaks), O111 (1 outbreak), O26:H11 (1 outbreak), O157:H7 and O121 (1 outbreak).

During 2007–2012, a total of 81 outbreaks associated with nonpasteurized milk were reported from 26 states. These outbreaks resulted in 979 illnesses and 73 hospitalizations. No deaths were reported. The causative agent was reported for all outbreaks. Of the 78 outbreaks with a single etiologic agent, *Campylobacter* spp. was the most common pathogen, causing 62 (81%) outbreaks, followed by Shiga toxin–producing *E. coli* (13 [17%]), *Salmonella* *enterica* serotype Typhimurium (2 [3%]), and *Coxiella burnetii* (1[1%]) (Figure 1). Three outbreaks were caused by multiple pathogens (Figure 1). The number of outbreaks increased from 30 during 2007–2009 to 51 during 2010–2012. During 2007–2009, outbreaks associated with nonpasteurized milk accounted for ≈2% of outbreaks with an implicated food; during 2010–2012, this percentage increased to 5%. The number of outbreaks of *Campylobacter* spp. infection also increased, from 22 during 2007–2009 to 40 during 2010–2012 (Figure 1).

**Figure 2:**
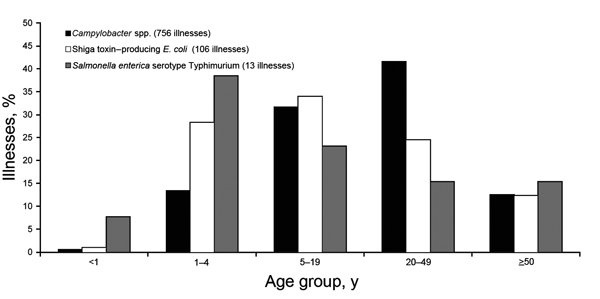
Percentage of patients affected by outbreaks associated with nonpasteurized milk, by age and etiologic agent, United States, 2007–2012.

Information about the age of patients was available for 78 outbreaks (Figure 2). For 59% of outbreaks, at least 1 patient <5 years of age was involved (Figure 2), and 38% of illnesses caused by *Salmonella* and 28% of illnesses caused by Shiga toxin–producing *E. coli* were in children 1–4 years of age (Figure 2).

How milk was obtained was reported for 68 (84%) outbreaks. Nonpasteurized milk was obtained from dairy farms (48 [71%] outbreaks), licensed or commercial milk sellers (9 [13%]), cow- or herd-share arrangements (8 [12%]), and other sources (3 [4%]) (Table). Of the 81 outbreaks, 66 (81%) were reported from states where the sale of nonpasteurized milk was legal in some form: Pennsylvania (17 outbreaks), New York, Minnesota (6 outbreaks each), South Carolina, Washington, and Utah (5 outbreaks each) (Table). A total of 15 (19%) outbreaks were reported in 8 states in which sales were prohibited (Table). Among these outbreaks, the sources of nonpasteurized milk were reported as a dairy farm (6 outbreaks), cow or herd share (4 outbreaks), and unknown (5 outbreaks) (Table).

**Table:** Source of milk in outbreaks associated with nonpasteurized milk, by legal status of state sales, United States, 2007–2012*

Legal status of raw milk sales (no. states)	Source where milk was purchased or obtained†						
	State (no. outbreaks)	Dairy farm	Licensed or commercial milk seller	Cow or herd share	Other‡	Not reported	Total
Allowed on farms and at retail stores separate from farms (legal, 12 states)	Pennsylvania (17), Washington (5), Utah (5), South Carolina (5), California (3), Idaho (1), Arizona (1), Connecticut (1)	21	7	1	3	6	38
Restricted to farms (legal, 13 states)	Minnesota (6), New York (6), Wisconsin (3), Kansas (2), Massachusetts (1), Nebraska (1)	14	2	1	0	2	19
Allowed on farm and at retail stores if standards met (legal, 1 state)	Vermont (4)	4	0	0	0	0	4
Only at farmer's markets (legal, 2 states)	Missouri (2)	2	0	0	0	0	2
Prohibited but allows cow or herd share (1 state)	Colorado (3)	1	0	2	0	0	3
Prohibited, including cow or herd share (illegal, 20 states)	Ohio (4), Michigan (4), North Dakota (2), Iowa (1), Indiana (1), Georgia (1), Alaska (1), Tennessee (1)	6	0	4	0	5	15
On-farm sales allowed only from farms with 2 producing cows, 9 producing sheep, and/or 9 producing goats (legal, 1 state)	0	0	0	0	0	0	0
Total		48	9	8	3	13	81

*Data for this analysis were downloaded on June 6, 2013. †Cow milk in 77 outbreaks, goat milk in 4 outbreaks. ‡Bed and breakfast lodging (1 outbreak), flea market (1 outbreak), raw milk buying club (1 outbreak).

## Conclusions

Within this 6-year period, the number of outbreaks associated with nonpasteurized milk increased. The number of outbreaks caused by *Campylobacter* spp. nearly doubled. The average number of outbreaks associated with nonpasteurized milk was 4-fold higher during this 6-year period (average 13.5 outbreaks/year) than that reported in a review of outbreaks during 1993–2006 (3.3 outbreaks/year) (*4*). This increase was concurrent with a decline in the number of states in which the sale of nonpasteurized milk was illegal, from 28 in 2004 to 20 in 2011 (*7*–*9*) and with an increase in the number of states allowing cow-share programs (from 5 in 2004 to 10 in 2008) (*8*,*9*). The decision to legalize the sale of nonpasteurized milk or allow limited access through cow-share programs may facilitate consumer access to nonpasteurized milk (*5*). The higher number of outbreaks in states in which the sale of nonpasteurized milk is legal has been reported elsewhere (*4*).

The legal status of nonpasteurized milk sales in 1 state can also lead to outbreaks in neighboring states. In a 2011 outbreak of *Campylobacter* spp. infections associated with nonpasteurized milk in North Carolina, where sales of this product were prohibited, milk was purchased from a buying club in South Carolina, where sales were legal. Another outbreak of *Campylobacter* spp. infection in 2012 implicated nonpasteurized milk from a farm in Pennsylvania, where sales are legal; cases from this outbreak were reported from Maryland, West Virginia, and New Jersey, all of which prohibit sale of raw milk (*10*). All patients residing outside Pennsylvania had traveled to Pennsylvania to purchase the milk (*10*).

Outbreaks associated with nonpasteurized milk continue to pose a public health challenge. Legalization of the sale of nonpasteurized milk in additional states would probably lead to more outbreaks and illnesses. This possibility is especially concerning for vulnerable populations, who are most susceptible to the pathogens commonly found in nonpasteurized milk (e.g., children, senior citizens, and persons with immune-compromising conditions). Public health officials should continue to educate legislators and consumers about the dangers associated with consuming nonpasteurized milk; additional information can be obtained at http://www.cdc.gov/foodsafety/rawmilk/raw-milk-index.html. In addition, federal and state regulators should enforce existing regulations to prevent distribution of nonpasteurized milk.
